# The Role of Surface Acting in the Relationship between Job Stressors, General Health and Need for Recovery Based on the Frequency of Interactions at Work

**DOI:** 10.3390/ijerph19084800

**Published:** 2022-04-15

**Authors:** Giulia Sciotto, Francesco Pace

**Affiliations:** 1 Department of Psychology, Educational Sciences and Human Movement, University of Palermo, 90128 Palermo, Italy; giulia.sciotto@unipa.it; 2 Department of Economics, Business and Statistics, University of Palermo, 90128 Palermo, Italy

**Keywords:** emotional labor, surface acting, health, need for recovery, wellbeing, stress

## Abstract

The aim of the study was to verify whether the frequency of face-to-face interactions with the public at work can reveal differences in how people react to emotional regulation demands. In particular, we investigated the mediating role of surface acting (a strategy of dealing with emotional dissonance) in the relationship between two typical job stressors (workload and mental load) and two outcomes closely related to work-related well-being: employees’ general health and the need for recovery. Prior studies investigating the detrimental effects of emotional dissonance mostly focused on service workers. However, in light of a survey conducted by the European Agency for Safety and Health at Work (2016) highlighting the growing psycho-social risk constituted by intense human interactions in the workplaces, even in unexpected categories of workers, we hypothesize that emotional demands may also be a concern for those who do not specifically interface with clients as part of their job duties. The results of the multi-group analysis of front-office (*N* = 734) and back-office (*N* = 436) Italian workers showed that surface acting fully mediates the relationship between workload and general health among back-office workers, while it only partially mediates this relationship among front-office workers. Furthermore, surface acting is positively associated with the need for recovery and negatively with general health, with higher values for back-office workers. The findings support the hypothesis that the emotional demands are not only a service worker issue and highlight the need to address emotional regulation strategies to enhance the quality of life in and outside the workplace for all employees.

## 1. Introduction

Work characteristics can directly influence employees’ psychological health and well-being [1]. Extended exposure to job demands such as work overload, time pressure and emotional demands may lead to exhaustion, job dissatisfaction and burnout symptoms [2,3], with heavier consequences if employees’ resources are already overtaxed [1,4], for example, by the request to display only certain emotions, irrespective of how they really feel [5]. In fact, research on work-related stress has shown that emotional stressors are predictors of higher levels of exhaustion and other adverse consequences for the mental and physical health of workers compared to other types of job stressors [1,2].

Many jobs that involve interactions with others require control over one’s emotions, for example, to improve customer satisfaction, appear competent, influence others or avoid unduly influencing others. The emotional demands that need to be faced may be internal (for example, from co-workers, supervisors, managers) or external (from clients, customers, patients, pupils, and so on); in both cases, individuals are expected to feign an emotion or an attitude that is congruent with the values and norms of their organization or job role, even when these differ from their true emotions [6]. In fact, employees may not always feel the emotions that they have to convey in a given work situation. When dealing with a patient who is impatient or a customer that makes unreasonable requests, individuals may find it difficult to maintain a friendly expression, or to appear positive or neutral, for example, when reacting to human suffering [7]. Depending on their emotional regulation competencies and their actual emotional state, adhering to organizational or contextual emotional-display rules can generate emotional dissonance, that is, a persistent and structural discrepancy between the emotions that need to be displayed and the emotions that are truly felt [8]. This has been widely reported as one of the most influential and distressing job demands [9,10,11,12,13,14], with the potential to lead to alienation from one’s own authentic emotions and cause psychological strain [15].

To date, the effects of emotional dissonance have mainly been studied in service-sector employees, since service-sector organizations frequently attempt to deliver high-quality service through the regulation of employees’ emotional expression at work [16]. In doing so, individuals’ autonomy over their emotional display is severely compromised, and a natural predisposition towards emotional dissonance develops. For example, employees who constantly interact with clients are often asked to smile cheerfully, even though they do not feel happy. In fact, the emotional state required by the organization, unlike the actual emotional state of individuals, does not change several times throughout the day. Thus, regardless of their true emotions, clerks and call center agents are always expected to be friendly and helpful, health service workers are always expected to be nurturing and reassuring, counter operators are expected to be sober, and so on [7,11]. Feigning happiness or enthusiasm proved to be particularly stressful [12] and common [17]. Happiness, or a smiling expression in general, is the most expected positive emotion, as it is linked to positive impressions, especially when working with the public [8,16]. Happiness is feigned more frequently than any other emotion in the workplace, not only when it is clearly required by the job role, but also during normal interactions with co-workers [17]. In some professions, showing happiness is an indicator of competence, professionalism, and mastery of emotional regulation strategies, as a sign of self-control capacity [12,17]. For example, teachers report frequently faking their enthusiasm, because behaving enthusiastically is believed to increase students’ interest and motivation [12]. Moreover, service employees might engage in the management of their emotional manifestation to ensure the customer’s perception of a high-quality service, even without an explicit request or monitoring actions by the organization [13]. This is a sign that these work-related emotional rules may be part of the collective knowledge and may act implicitly.

In response to the job-specific emotional display requirements, employees can perform two strategies of emotion regulation: surface acting and deep acting [8]. In surface acting, employees alter their displayed feelings to convey a false emotional display that is faithful to the organization’s norms, while their inner feelings remain unchanged. Surface acting, therefore, is characterized by a dissonance between the displayed and felt emotions, a state usually associated with feelings of inauthenticity [4]. In contrast, deep acting works on modifying arousal or cognitions to create a state of emotional consonance between the displayed and felt emotions [4]. Research widely suggests that surface acting has the most detrimental effect on employees’ well-being [5,13,18], job satisfaction and emotional exhaustion [6,19,20]. The lower the authenticity of the surface-acting, the higher its association with poorer emotional and physical outcomes for individuals [5,21].

The negative impact of surface acting on well-being can be explained in several ways. First, emotional demands may be perceived as an unjustified restriction of personal autonomy, especially when employees are disrespected [22]. Second, if employees are not used to interacting with clients or customers, the act of faking emotions that are not actually felt can represent a stressor that they are not properly trained to deal with [14]. Third, the act of aligning felt and required emotions requires a large cognitive effort, and the regulatory costs implied by this effort may result in strain [18,20,21,23]. Therefore, similarly to other types of self-control, emotional control depletes mental resources, and it is likely that when employees experience emotional dissonance, they will also experience a higher level of strain [10]. Moreover, when people frequently exert this type of self-control without being able to replenish their depleted resources, psychological well-being is proposed to be considerably impaired [4,9,11].

Under normal conditions, subjects are able to recover their energy at the end of the working day and recharge their emotional and attentional resources for the next working day. In cases of insufficient recovery, however, they must make additional and compensatory efforts in order to perform well and successfully complete their tasks the next workday [24]. If recovery continues to fail, already overworked employees might have to struggle to concentrate and juggle various tasks. Impaired ability to concentrate contributes to an increase in the need for recovery [25] and coping skills in order to manage workers’ daily job demands [23].

Recovery from work refers to the process of reducing or eliminating the physical and psychological strain symptoms that were caused by job demands and stressful events at work [26]. It includes the ability to mentally disengage from work during time off-the-job. Unsuccessful recovery is related to a reduced ability to concentrate, difficulty relaxing during leisure time, difficulty detaching from work-related thoughts at the end of a workday, tiredness, more time being required to restore energy, poor well-being, and health problems [25,27,28]. The more intense the working day, the longer it takes to relax and restore energy levels in the evening. People who reported particularly stressful working days had higher levels of adrenaline in the evening and reported sleep disorders the next morning, along with having a greater perception of fatigue and lower levels of well-being than other groups [27]. For example, a study on the effects of surface and deep acting on the health-impairment process [23] showed that surface acting was directly involved in predicting employees’ need for recovery at the end of their working day. Higher levels of surface acting during work were associated with lower energy levels at bedtime and an impaired ability to relax during leisure time [23]. Failing to recover from the workday also prevented individuals from engaging in other equally necessary (and fatiguing) activities, such as household or family obligations [27,29]. This could lead to a vicious circle, where work-induced fatigue increases difficulties relaxing, which, in turn, affects individuals’ private life and serves as a risk factor for mental health [4,29,30].

In line with the health-impairment process of the Job Demands–Resources Model (JD-R) [1], the inability to efficiently manage work activities as a consequence of unrecovered fatigue can trigger a further depletion of resources and have negative long-term results. In fact, emotional dissonance may not be stressful in itself, but can become increasingly dangerous for employees’ well-being at work when combined with other job stressors that require increased effort [4]. When people invest their energy in meeting their workload and further invest it in fulfilling emotional requirements, this act may result in excessive energy investments, and is likely to exhaust employees [5]. The more the employees’ resources are depleted by the high rhythm of work and the concentration needed to carry out the job tasks, the more employees’ may need to expend their energy and effort to also control and regulate their emotions. Therefore, job demands can have a direct effect on employees’ well-being and need for recovery [1], and an indirect effect through surface acting, which has been widely associated with work strain and psychological distress across different work samples [6,13,18,20,23], and can, therefore, worsen the relationship between job demands and work-related outcomes [4]. In other words, surface acting can function as a mediator between work characteristics and fatigue, on the one hand, and psychophysical health, on the other [5].

Hence, we hypothesize that:

**Hypothesis** **1** (**H1**). *Surface acting mediates the relationship between job stressors (workload and mental load) and need for recovery.*

**Hypothesis** **2** (**H2**)**.** *Surface acting mediates the relationship between job stressors (workload and mental load) and general health perception.*

In the present study, the aim is to test the hypothesized relationships between these variables among both front-office and back-office workers. To our knowledge, the role of emotional demands as job stressors for workers who deal less frequently with the public has not yet been verified. Prior research investigating the effects of emotional dissonance has focused on service-sector employees, since emotional requirements and their consequences are rightly perceived as more relevant in occupations where workers must directly deal with other people as a mandatory part of their job duties. However, the hypothesis that emotional dissonance might also be a concern for back-office workers is supported by a survey conducted by the European Risk Observatory, where respondents were asked about the presence of risk factors in their establishments. The most frequently identified risk factor was having to deal with difficult customers, pupils, or patients (58% of establishments), which was reported as being even more stressful than physical demands [31]. The respondents belonged to different professional categories, including back-office workers. For this type of workforce, however, the lack of literature regarding the presence or the weight of coping with emotional job demands leads to their training and professional development needs relating to emotional labor being overlooked. The lack of adequate training on how to cope with emotional demands, in turn, may compromise the well-being of individuals at work and, consequently, their overall health and functioning in daily life.

Hence, we hypothesize that:

**Hypothesis** **3** (**H3**)**.** *The type of job (front or back-office, based on the frequency of face-to-face interactions) can reveal differences in how these job stressors are associated with the need for recovery and the general health perception.*

## 2. Materials and Methods

A multi-group analysis was used to simultaneously verify the mediating role of surface acting in the relationship between job stressors (workload and mental load), need for recovery and general health perception among front-office (*N* = 734) and back-office (*N* = 436) workers. Descriptive data analysis, Pearson correlations and Cronbach’s alpha coefficients were tested using SPSS 27 (IBM, Armonk, NY, USA). Confirmatory factor analysis (CFA) and Structural Equation Modeling were carried out with Mplus 8 (Muthén and Muthén, Los Angeles, CA, USA). Measurement invariance was tested by comparing the two samples of back-office workers and front-office workers. First, separate confirmatory factor analyses (CFA) were performed for the two samples, and the goodness-of-fit to the data was evaluated. Then, the multiple-group confirmatory factor analysis (MGCFA) was used to assess the following measurement invariance: configural (no equality constraints on parameters between groups), metric (equality constraints on factor loadings), scalar (equality constraints also on thresholds), and strict (equality constraints also on residual variances). These additional constraints can be maintained if the decrease in Comparative Fit Index (CFI) between the adjacent nested models is less than 0.01 [32]. Once support for measurement invariance was obtained, the structural models were examined. The goodness of fit of the two structural models for the two samples and the structural model across groups was examined, and the adequacy of the data was confirmed. Finally, to assess the significance of the indirect effects, the bootstrapping method with 5000 replications was used. The estimation method was maximum likelihood (ML), and the following criteria were used to evaluate the goodness of fit: χ^2^ likelihood ratio statistic, Tucker–Lewis index (TLI), comparative fit index (CFI) and the root mean square error of approximation (RMSEA) with associated confidence intervals. RMSEA values lower than 0.08 and CFI and TLI values greater than 0.90 indicated acceptable fit [33].

### 2.1. Participants

The research population for this study was a convenience sample of 1170 Italian workers, contacted through emails and invited to fill out an online questionnaire, along with the informed consent documents. Among the socio-demographic questions in the questionnaire, we asked subjects to categorize themselves as front-office workers or back-office workers, based on how many interactions with the public their work required. Overall, 55% of the subjects were male and 45% were female; mean age was 43.8 years old (SD = 10.8), with a range between 20 and 67 years old. The sample was made up of 37% of workers who performed back-office functions (little contact with the public) and 63% of workers who performed front-office functions (frequent interactions with the public). The back-office sample consisted of 70% public-sector administrative staff and 30% private-sector employees (Human Resources, bank services, industry workers). The front-office sample consisted of 9% public-sector administrative staff, 18% private-sector employees (Human Resources, bank services, industry workers), 10% armed forces workers, 18% teachers, 15% salesclerks, 26% healthcare workers, and 4% call-center operators.

### 2.2. Measures

The Italian version of the Questionnaire on the Experience and Evaluation of Work (QEEW) [34] was used to measure workload, mental load and need for recovery. All items were assessed using a 4-point Likert scale, from (1) “never/absolutely no” to (4) “always/absolutely yes”; a higher score indicates a higher presence of the construct. The *workload* scale evaluates the pace and the amount of work to which the worker is subjected during a regular workday. Examples of the items include: “Do you have to work extra hard in order to complete something?”, and “Do you work under time pressure?”. Cronbach’s alpha for the scale in this study was 0.70. The *mental load* scale measures the degree of concentration and attention required from the worker during the execution of the job. An example of an item is: “Does your work demand a lot of concentration?”. Cronbach’s alpha for the scale in this study was 0.72. The *need for recovery* scale evaluates the feeling of physical and mental fatigue resulting from the working day. Examples of the items include: “I find it difficult to relax at the end of a working day”, and “Because of my job, at the end of the working day I feel rather exhausted”. Cronbach’s alpha for the scale in this study was 0.83.

The perception of surface acting was assessed using the *surface acting* scale of the Emotional Labour Scale [19]. For convenience, the affirmative items of the original scale were transformed into questions. All items were assessed using a 4-point Likert scale from (1) “never/absolutely no” to (4) “always/absolutely yes”; a higher score indicated a higher presence of the construct. An example of the items is as follows: “In certain circumstances of your work do you have to hide your true feelings?”. Cronbach’s alpha was 0.71.

Health perception was measured using the 12-item version of the General Health Questionnaire (GHQ-12) [35]. The first 6 items have a positive value and investigate concentration, coping and decision-making abilities. Six sensations were described, and subjects were asked to report how often they have experienced these sensations in the last four weeks on a 4-point Likert scale (from 1 = “much less than usual” to 4 = “much more than usual”). The remaining 6 items assess negative feelings such as tension, unhappiness, the presence of worries or sleep problems. The frequency is rated on a 4-point Likert scale (from 1 = “No” to 4 = “much more than usual”). The scores of the last 6 items were reversed, so that the overall score of the questionnaire indicates the level of perceived well-being. Cronbach’s alpha was 0.85.

## 3. Results

Table 1 shows means, standard deviations and correlations between the study variables in both samples.

Prior to conducting multiple-group analyses to test our hypotheses, we examined the goodness-of-fit values of the CFA models separately for both samples. The results are shown in Table 2.

**Figure 1:**
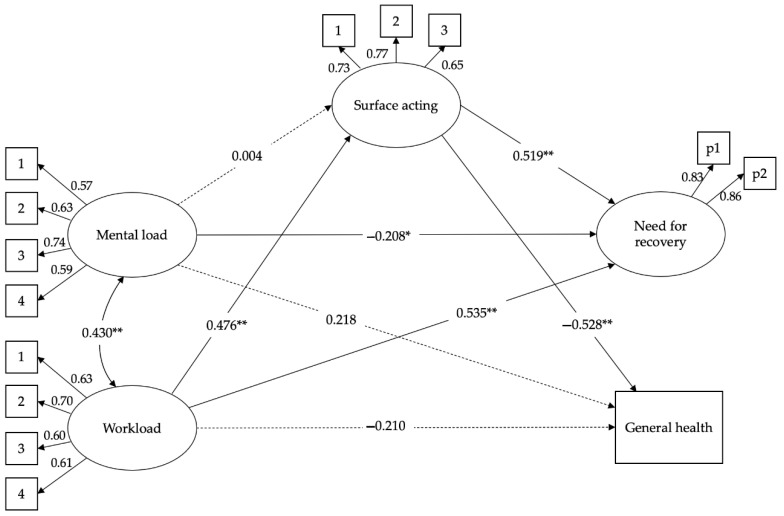
The structural model for the back-office sample (*N* = 436). * *p* < 0.05 **, *p* < 0.01.

**Figure 2:**
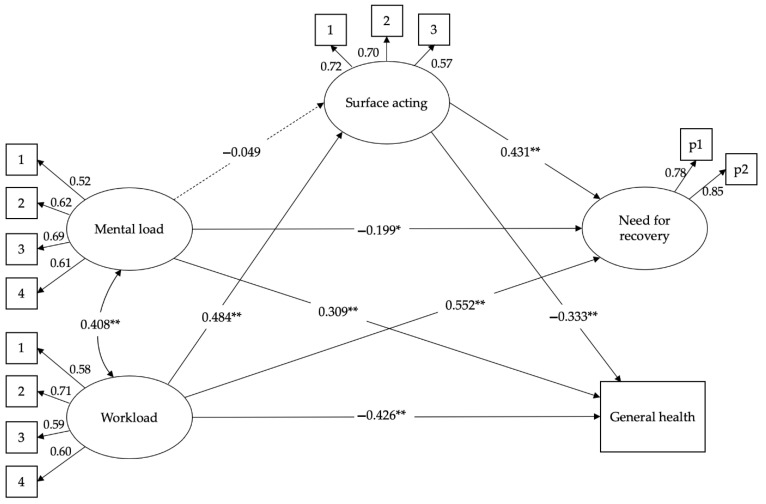
The structural model for the front-office sample (*N* = 734). * *p* < 0.05 **, *p* < 0.01.

Both CFA models show good fit. Each indicator had statistically significant (*p* < 0.001) factor loadings on its assigned construct. Table 2 also shows the results of analyses for measurement invariance testing across the two groups. The four increasingly constrained models testing measurement invariance also show good fit indices, and the decrease in CFI between adjacent models was less than 0.01. Thus, the results indicate that there is, overall, good evidence of there being no substantial item bias in the data, and measurements can be meaningfully compared across groups. As shown in Table 2, both structural models testing study hypotheses yielded a good fit to the data. Moreover, results from the subsequent comparison of single analysis across both groups still show a good fit, thus supporting an invariant pattern of relationships among variables across the back-office and the front-office workers samples. Structural models for both samples are graphically presented in Figure 1 and Figure 2.

As represented in Figure 1 and Figure 2, the two models show some differences, validating hypothesis 3. In the back-office workers’ model, workload is positively related to surface acting (β = 0.476 **, se = 0.07) and need for recovery (β = 0.535 **, se = 0.07), and negatively associated with general health perception (β = −0.210, se = 0.14). However, the latter direct effect is not significant, as the relationship between workload and general health perception is fully mediated by the surface acting (see Table 3 for indirect effects). In the front-office model, workload is positively related to surface acting (β = 0.484 **, se = 0.07) and need for recovery (β = 0.552 **, se = 0.07), and negatively associated with general health perception (β = −0.426 **, se = 0.08). In this case, however, the direct effect of workload on worsening the general health perception was significantly greater. Surface acting mediates both the relationships between workload and need for recovery and general health perception. Hypotheses 1 and 2 are confirmed, but only regarding the workload variable. In fact, in both models, the relationship between mental load and surface acting is not significant; therefore, surface acting has no mediation role. Table 3 reports all the indirect effects as evaluated by the bootstrapping analysis.

The results confirm the indirect effect of surface acting in the relationship between workload and general health perception. In the back-office sample, the mediation is complete. Surface acting acts as a mediator in the relationship between workload and need for recovery. This is valid for both samples.

The second difference that stands out between the two samples is the role of mental load. In the back-office workers’ model, the relationship between mental load and general health perception is not significant (β = 0.218, se = 0.12), while this is not the case for the front-office sample (β = 0.309 **, se = 0.07). In both models, however, mental load is negatively associated with the need for recovery (β = −0.208 *, se = 0.08 for the back-office sample; β = −0.199 *, se = 0.07 for the front-office sample). This type of job demand does not seem to constitute a burden that aggravates work stress, but rather serves as a motivational factor.

## 4. Discussion

In this study, we examined how some work stressors (workload, mental load, and surface acting) are associated with general health perception and the need to recover physical and mental energies at the end of the working day. In doing so, we questioned whether the different frequency rates of face-to-face interactions might reveal differences in how these job stressors are linked to perceived health and fatigue.

The results show that surface acting mediates both the relationships between workload and need for recovery and general health perception. For the front-office sample, surface acting and workload are equally demanding. Coping with surface acting is linked to psychological costs, which manifest in higher levels of need for recovery and an impaired perception of psychophysical health. Furthermore, when dealing with high levels of workload, the adverse effect of emotional labor is amplified. These findings might support the notion that coping with workload and surface acting simultaneously depletes individuals’ resources [4,9,11,25].

In the back-office sample, however, the mediation of surface acting in the relationship between workload and general health perception is complete. Moreover, in this sample, surface acting is more positively related to need for recovery and more negatively to general health perception compared to the front-office sample. In fact, the act of feigning emotions seems to be more stressful for workers who are not used to interactions with the public (customers, patients, students, etc.). This leads to an important conclusion. The well-being of individuals in the workplace is made up of innumerable factors, most of which are inextricably linked [1,2,4]. Job experiences affect people’s well-being even when they return home [26]. A widely recognized stressor in research is emotional dissonance [8,15]. This has been extensively studied in service organization workers and its performance has been proven to be detrimental to employees’ health [9,16], while it has been widely overlooked for back-office workers. However, an in-depth study may be particularly important for this type of worker. While this study cannot delineate causal relationships, its findings can be used as a starting point for delving into the role that interactions with the public play in samples of workers who are not used to such interactions. In fact, the finding that, for subjects who perform back-office activities that do not involve interactions with the public, surface acting is the most impactful antecedent of impaired health, even more than perceived workload and mental load, may be a major theoretical contribution to the work stress and occupational well-being research field. This could demonstrate that having to express positive emotions such as interest or courtesy when no specific emotion is being felt, or even when a negative emotion is being experienced, is an unbearable demand that transcends the type of profession. As illustrated above, emotional demands lead to a decrement of resources and, therefore, increase the individuals’ fatigue. Increases in the levels of work-related fatigue have repercussions for people’s general quality of life in. Those who report higher levels of fatigue associated with work also report difficulties in relaxing, engaging in leisure activities and even spending quality time with their families [26,28]. Therefore, it can be concluded that the burden of emotional labor can generate chain reactions that lead to individuals feeling too tired to take corrective actions that could be beneficial to his or her well-being. Underestimating the danger of this vicious circle puts people’s quality of life at risk.

Finally, the role of mental load in this study proved to be counterintuitive. Acting as a resource and not a stressor, it did not worsen the perception of well-being, nor did it lead to an increase in the need for recovery. The literature has already highlighted how the effect of job demands may vary based on whether the demands are interpreted as a hindrance or a challenge [14,36,37]. In this case, the presence of work activities that require high levels of concentration, attention and memory may lead to a positive perception of what is being achieved in the workplace. The need to exert concentration and carefulness may be linked to the perception of doing something important. Therefore, the ability to perform these job activities could play a fundamental role in workers’ well-being in the workplace, as it may be connected to perceptions of competence and mastery. The logical step that leads us to think that mental load is linked to greater motivation and interest in work is not justified by the research design, but it could be a future evolution.

### Limitations and Future Research

The limitations of the study stems from its cross-sectional nature, meaning that no reliable conclusions can be drawn regarding the causal direction of effects. A longitudinal research design should be implemented to confirm or disprove what has emerged to date. We also recommend exercising caution when generalizing the results. In fact, future research observing employees in diverse professions should use larger samples to verify to what extent our findings can be generalized. Another limitation stems from the employment of self-report data only. Future studies should also examine other variables that may affect the need for recovery and the general health perception. Consistent with the analysis of the literature, it is possible that other intervening variables, such as work–life balance and the level of job control, have an influence on the perception of work stress. This study, however, could be a valuable preliminary study that responds to the lack of literature concerning non-service professions, underlining that the reasons for back-office workers’ stress can and should be investigated with the same accuracy that is reserved for all other professions.

## 5. Conclusions

Studies on work-related stress highlight the importance of reasoning regarding emotional load and the expression of emotions in all workplaces. The focus of attention is on both external relationships, which are typical of service companies, and internal relationships, where emotional control and a “relational grammar” far from that of authentic relationships are increasingly required. Undoubtedly, there are categories of workers that are constantly subjected to emotional dissonance. However, back-office workers may often find themselves in a situation where they have to display certain emotions, which may or may not be consistent with their true self. The lack of habitual contact with users and, consequently, the lack of training when interacting with the public means that many workers face great difficulties when, forced to interact with others, they are also forced to show emotions that they may not really feel. This, to an even greater extent than merely quantitative workload, may affect their well-being and their tiredness at the end of the working day. It is conceivable that the back-office workers have had fewer previous requests regarding social interaction (both internally, towards their co-workers, and externally) in the workplace (for example, they may have participated in fewer meetings or may never have worked in a team), and that the evolution of organizations has led to workers being increasingly exposed to the psycho-social risks associated with the need to maintain stable control over their perceived emotions. Therefore, while front-office workers are often adequately trained and know useful strategies for balancing emotional detachment, in which they maintain an assertive position with both external and internal clients, it is possible that the same attention has not been reserved for back-office workers.

This study can have both practical and theoretical value. Regarding the theoretical contributions, this study attempts to fill a void in the literature, which has overlooked the systematic assessment of emotional demands among non-frontline workers to date. The simultaneous evaluation of the same model on two different samples of workers highlighted differences in terms of the weight of the emotional regulation rules and their repercussions for physical and mental fatigue. Acknowledging the fact that emotional dissonance is a stressor shared by every type of worker is essential for structuring corrective and preventive interventions. Another important theoretical contribution concerns the confirmation of the difference between hindrance and challenge stressors. In this case, the assumption that the need for attention and concentration required by a job is a burden has been proved wrong. This finding can be considered further evidence of the crucial importance of work-related stress assessments, since only exact and in-depth knowledge of workers’ perceptions about the characteristics of their work allows for targeted and, therefore, effective interventions. Regarding the practical implications, the results suggest that it may be useful to encourage greater harmonization of Human Resources Management practices (from selection procedures, to onboarding and ongoing training) regarding strategies for managing emotions in the workplace. For example, frequent assessments of work-related stressors can be useful in tracking back-office workers’ distress levels over time. These assessment procedures must also include sections relating to emotional labor for those who carry out back-office work, so that it is no longer taken for granted that workers with fewer interactions with the public do not suffer equally from emotional dissonance. Indeed, precisely because they are less accustomed, and perhaps less temperamentally inclined, these workers need specific training on how to cope with the need to hold back their true self in favor of socially and organizationally accepted emotions. Moreover, it would also be useful to reflect on the need to create a communication model based on authenticity and assertiveness. Therefore, alongside training on emotion regulation strategies, organizational interventions aimed at enhancing communication and relational skills would be recommended. Given the importance of the perception of authenticity and the alignment between the emotions that are felt and emotions that are shown, neglecting an assessment of the perceived quality of internal relationships risks generating distress in the workplace, with direct consequences for the psychophysical well-being of workers.

In conclusion, it would be desirable if organizational interest in this topic continued, and if training initiatives for emotional regulation strategies, while remaining anchored to the real emotional demands of the various jobs, were not neglected for back-office work. It is necessary to remember that the negative repercussions of work stressors are not limited to working life. When workers have to perform something that they are not adequately trained or inclined to do, the risk affects not only their job performance, but also their mental and physical health, their psychological functioning, and their ability to relax and participate fully in their family life. The goal should be the creation of work contexts that protect the well-being of all individuals in the workplace.

## Figures and Tables

**Table 1:** Means, standard deviations, and correlations among study variables (back-office sample *N* = 436; front-office sample *N* = 734).

Variable	Mean	SD	1	2	3	4	5
1. Workload	2.89 (2.77)	0.87 (0.92)	1	0.328 **	0.242 **	0.640 **	−0.269 **
2. Mental Load	3.41 (3.42)	0.69 (0.74)	0.305 **	1	0.210 **	0.225 **	−0.069
3. Surface Acting	2.34 (2.41)	0.97 (0.97)	0.276 **	0.210 **	1	0.287 **	−0.354 **
4. Need for Recovery	2.28 (2.20)	0.94 (0.93)	0.668 **	0.220 **	0.476 **	1	−0.355 **
5. General Health	2.36 (2.39)	0.77 (0.76)	−0.293 **	−0.033	−0.442 **	−0.415 **	1

Correlations below the diagonal are for the back-office sample and correlations above the diagonal are for the front office sample. Mean and SD for the front office sample are in parentheses. ** *p* < 0.01.

**Table 2:** Goodness-of-fit values of CFA models, multi-group test for measurement invariance, and the structural models testing the study hypotheses (ML estimation; back-office sample *N* = 436; front-office sample *N* = 734).

Models	Model Fit						
	χ^2^	df	CFI	TLI	RMSEA (90% CI)	ΔM	ΔCFI
Model for back-office	242.664	113	0.944	0.933	0.051 (0.042–0.060)		
Model for front-office	300.177	113	0.945	0.932	0.049 (0.042–0.055)		
M1: Configural	420.529	225	0.932	0.917	0.055 (0.046–0.063)		
M2: Metric	441.323	238	0.929	0.919	0.054 (0.046–0.062)	M1-M2	0.003
M3: Scalar	468.849	250	0.924	0.917	0.055 (0.047–0.062)	M2-M3	0.005
M4: Strict	502.014	266	0.918	0.916	0.055 (0.048–0.042)	M3-M4	0.006
Structural model for back-office	163.852	68	0.944	0.926	0.057 (0.046–0.068)		
Structural model for front-office	187.711	68	0.952	0.934	0.050 (0.041–0.058)		
Structural model across groups	393.162	152	0.944	0.933	0.052 (0.046–0.058)		

**Table 3:** Indirect effects using bootstrapping with 5000 replications (ML estimation; back-office sample *N* = 436; front-office sample *N* = 734).

Indirect Effects	Est.	S.E.	95% CI
Back-office workers sample			
Workload → Surface Acting → Need for Recovery	0.248 **	0.044	0.179, 0.322
Workload → Surface Acting → General Health	−0.257 **	0.054	−0.353, −0.177
Front-office workers sample			
Workload → Surface Acting → Need for Recovery	0.216 **	0.038	0.155, 0.282
Workload → Surface Acting → General Health	−0.166 **	0.035	−0.226, −0.133

**, *p* < 0.01.

## Data Availability

The data presented in this study are available on request from the corresponding author.
